# Alterations in the odor profile of plants in cultivar mixtures affect aphid host-location behavior

**DOI:** 10.3389/fpls.2023.1186425

**Published:** 2023-06-07

**Authors:** Alba Tous-Fandos, Jannicke Gallinger, Arnoud Enting, Lourdes Chamorro-Lorenzo, F. Xavier Sans Serra, Velemir Ninkovic

**Affiliations:** ^1^ Department of Evolutionary Biology, Ecology and Environmental Sciences, University of Barcelona, Barcelona, Spain; ^2^ Institut de Recerca de la Biodiversitat (IRBio), Universitat de Barcelona (UB), Barcelona, Spain; ^3^ Department of Ecology, Swedish University of Agricultural Sciences, Uppsala, Sweden; ^4^ Aeres University of Applied Sciences, Almere, Netherlands

**Keywords:** aphid host location, genotypic diversity, functionality, plant-plant interaction, plant odor cues, volatile organic compounds, wheat

## Abstract

The effect of cultivar mixtures on aphid control is attributed to the masking or alteration of host-preferred cultivar odor cues. However, the underlying physiological mechanism remains unclear. This study assessed alterations in the volatile emissions of wheat cultivars grown together (Florence-Aurora and Forment; Florence-Aurora and Montcada) and the consequences for the olfactory preference of aphids. Volatile organic compounds were collected from wheat plants grown in a laboratory under mixed or monoculture conditions and subsequently analyzed. The odor profiles of Florence-Aurora and Montcada were indistinguishable from each other. However, the odors of Florence-Aurora and Forment grown in monocultures differed significantly from those emitted by their mixture. The Florence-Aurora and Forment mixture induced plant physiological responses that affected the emission of single volatile compounds and, consequently, altered volatile organic compound ratios. English grain aphids (*Sitobion avenae*) were less attracted to the odors of Florence-Aurora and Forment when grown as a mixture than the combination of the odors from Florence-Aurora and Forment monocultures. Moreover, aphids preferred clean air over the odor from the Florence-Aurora and Forment mixture but preferred the odor from the Florence-Aurora and Montcada mixture over clean air. This study highlights the beneficial effects of intraspecific plant diversity on aphid control by altering plant odors in response to plant-plant interactions. The emission of less attractive odor cues consequently affects plant-aphid interactions; hence, less attractive odors are likely to impair aphid host-locating behavior. This effect was exclusive to certain cultivar mixtures, which supports the “right neighbor” concept.

## 1 Introduction

As sessile organisms, plants are constantly exposed to several threats, such as adverse ambient conditions, resource competition, and herbivore attacks; hence, they have developed highly sophisticated strategies to guarantee their survival. For instance, plant-plant interaction is a key mechanism for enhancing plant fitness in competitive scenarios, such as cultivar mixtures (Murphy and Dudley, 2009; Ninkovic et al., 2016). Such interactions between cultivars can lead to unfavorable (associational susceptibility) or favorable (associational resistance) associations for neighboring plants that influence their susceptibility to herbivorous insects (Barbosa et al., 2009).

The effect of intraspecific plant diversity on aphid control has been attributed to associational resistance. This suggests that combining the right cultivars reduces herbivorous damage to the host-preferred cultivar by inducing competition-related changes that affect herbivore feeding. These may include the production of anti-herbivore defenses, alteration of host plant quality, or interference in the host location of herbivores (Barbosa et al., 2009).

Furthermore, several studies have demonstrated that the benefits of genotypic diversity are cultivar-specific and depend on the interactions between the cultivar mixtures, leading to the “right neighbor” concept (Dahlin et al., 2018; Kheam et al., 2023). For instance, Ninkovic et al. (2002) tested the aphid control effect of barley cultivar mixtures in a field experiment using various pairs of barley cultivars; they demonstrated that only certain combinations decreased aphid acceptance.

Host selection in aphids is an extremely complex process that involves a variety of sensory and behavioral mechanisms (Powell et al., 2006). Previous studies have emphasized the importance of plant odor signals in aphid host identification, location, and acceptance (Pickett et al., 1992; Webster, 2012). Volatile organic compounds (VOCs) are detected via the antennal olfactory sensilla and can be used to locate host plants prior to settling to determine the quality of the phloem composition (Powell et al., 2006). Hence, understanding the role of plant olfactory cues in aphid host location behavior may lead to improved integrated pest management strategies.

Furthermore, it is well-documented that plants respond to neighboring volatiles, causing morphological and physiological modifications (Callaway and Walker, 1997; Ninkovic et al., 2016; Ninkovic et al., 2021). For instance, VOCs from herbivore-damaged plants can trigger defensive responses in neighboring undamaged plants (Glinwood et al., 2009; Midzi et al., 2022). Furthermore, VOCs from undamaged plants can induce physiological shifts in neighboring plants, increasing cultivar resistance to aphids or altering tritrophic interactions (allelobiosis) (Ninkovic et al., 2006; Dahlin et al., 2018; Kheam et al., 2023).

Previous studies have mostly focused on volatile interactions between cultivars and their implications for aphid acceptance (Ninkovic et al., 2002; Dahlin et al., 2018). Less is known about the complete plant-plant interactions when grown together, their physiological response, and their consequences on allelobiosis. Hence, this study sought to investigate these aspects. Additionally, gaining a deeper understanding of the physiological mechanisms underlying associational resistance may serve as a foundation for enhancing “right neighbor” combinations and, by extension, as a design tool for functional agrobiodiversity (Barbosa et al., 2009; Gaba et al., 2015; Borg et al., 2018).

Therefore, this study aimed to assess the influence of overall above- and below-ground wheat cultivar interactions, when grown together to simulate field conditions, on the mixture odor profile and its subsequent effect on aphid host location behavior. Two previously field-tested wheat mixtures with varying aphid control capacities were compared. We hypothesized that (i) genotypic diversity effects on aphid host location would be specific to the cultivars combined, (ii) the coexistence of cultivars in the mixture would induce changes in their volatile profiles, and (iii) aphids would prefer odor cues from monocultures over those from cultivar mixtures.

## 2 Methodology

### 2.1 Plants and insect material for the experiments

Three winter wheat cultivars were used in the experiments: the modern cultivar Florence-Aurora (*Triticum aestivum* L. *subsp. Aestivum)* and the traditional cultivars Forment (*Triticum turgidum* L. *subsp. Durum*) and Montcada (*Triticum aestivum* L. *subsp. Aestivum).* Seeds were supplied by farmers from the Gallecs Agroecological Union and technical personnel from the Gallecs Natural Interest Area Consortium in Catalonia, Spain. The three wheat cultivars were either grown as monocultures: Florence-Aurora (FA), Forment (FO), and Montcada (MO), or in two cultivar mixtures: 1:1 Florence-Aurora and Forment (FAFO) and Florence-Aurora and Montcada (FAMO). The selection of cultivars and mixtures was based on the farmers’ preferences, with Florence-Aurora being the principal cultivar, owing to its excellent bread-making qualities. Both mixtures have exhibited contrasting aphid control abilities in previous field studies (unpublished data). Florence-Aurora was treated as the principal cultivar because it is the most cultivated cultivar in the Gallecs Agroecological Union. Four wheat plants were planted together in plastic pots (13 × 13 × 23 cm) in potting soil (Hasselfors Garden P soil). The plants grew under controlled conditions in a growth chamber at 18–21°C with a 16/8-h light/dark cycle. Olfactometer experiments and headspace collections were conducted on 1-month-old wheat plants. The English grain aphid [*Sitobion avenae* (Fabricius)] was reared on oats (*Avena sativa* L. cv. Belinda) in multiclonal cultures in separate rearing chambers under identical conditions.

### 2.2 Aphid olfactory response

Aphid olfactory responses to different wheat odors were examined using a two-way airflow olfactometer, which consisted of two stimulus zones in which the odors were introduced and a central zone separating them. Airflow through the system was set to 180 ml min^-1^ measured with a flow meter at the arm inlets (Ninkovic et al., 2013). Plants used as odor sources were placed inside chamber cages directly connected to the olfactometer arms. An adult wingless *S. avenae* was carefully inserted in the middle of the olfactometer using a fine brush. After acclimatization for 10 min, the position of each aphid was recorded at 3 min intervals for 30 min. Each aphid was used only once and was regarded as a replicate. Olfactometers were cleaned with 70% ethanol between trials. Aphids that did not move after acclimatization were excluded from the analysis. The experiment was conducted in a dark room under artificial light (Osram FQ80W/840 HO Constant Lumilux Cool White (4000 K); Munich, Germany) at 60 µmol m^-2^ s^-1^ above the olfactometer to limit the influence of visual inputs. Further, the experiments were conducted on sunny spring days from 9:00 am to 4:00 pm. The average room temperature was 20°C and relative humidity was 40-50%.

**Figure 1:**
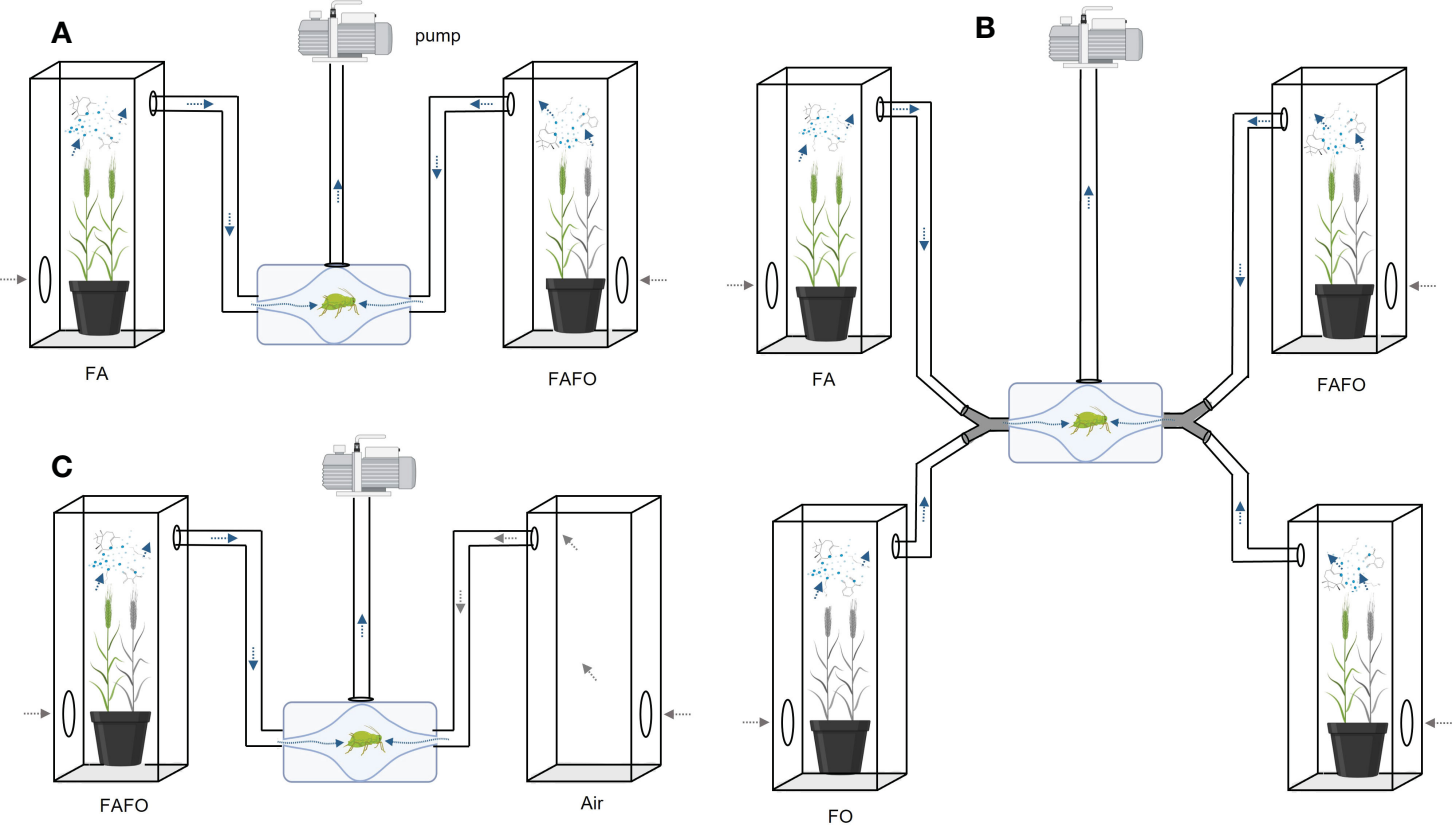
Schematic of the experimental design of the olfactometer assays. Pots with four wheat plants were placed in each cage connected to an olfactometer and a suction pump that was used to facilitate airflow from the plants through the olfactometer. An apterous adult aphid was placed in the middle of the olfactometer. **(A)** Representative single-pot comparisons between monocultures and their respective mixtures. **(B)** Representative comparison of two vs two pots. To compare mixed odors of monocultures (one cultivar per pot/cage) and their mixture (two pots/cages of mixtures), two cages were connected using Y-connectors to mix volatile cues before entering each olfactometer arm. **(C)** Schematic of the avoidance test. Aphid movement towards plant odors was compared with clean air. Wheat cultivars: FA, Florence-Aurora; FO, Forment; MO, Montcada.

First, we compared the monocultures with each other or their respective cultivar mixtures (FA vs. FO, FA vs. MO, FA vs. FAFO, FO vs. FAFO, FA vs. FAMO, and MO vs. FAMO; **Figure 1A**). Second, we investigated the olfactory responses of aphids to mixed odors from the monocultures. To mix the odor of plants from two pots, each pot was placed in a separate cage, but both cages were connected by a Y-connector to the same olfactometer inlet, resulting in a mixed odor of the two monocultures (FA + FO and FA + MO). Through this method, the odors of the two separately grown monocultures could be introduced on one side of the olfactometer and tested against clean air. Thus, two plants were used as one odor source. We offered this mixed odor simultaneously with the odors of the corresponding cultivars grown in a mixture (FA + FO vs. FAFO and FA + MO vs. FAMO). To equalize the number of plants (biomass), two pots of the cultivar mixtures were connected to an olfactometer, as described for the monocultures (**Figure 1B**). For the avoidance test, we compared cultivar mixtures (FAFO and FAMO) and mixed odors from monocultures (FA + FO and FA + MO) with clean air (**Figure 1C**). Each treatment comparison was replicated with 14–27 aphids. Data were analyzed using Wilcoxon matched-pair tests in R, version 4.1.1 (R Core Team, 2021).

### 2.3 VOC collection

To avoid volatile interactions between the plants, each pot with wheat plants was grown separately inside clear Perspex chamber cages (10 × 10 × 80 cm) (Ninkovic et al., 2002). Air was allowed to enter the forward chamber through an opening in the cage wall (7 cm in diameter), extracted through a tube attached to a vacuum tank, and vented outside the room using an electric fan. The airflow through the cages was 1.3 L min ^-1^. Volatiles were collected using a push-pull system. The upper part of the pot containing the four wheat plants was placed inside a polyethylene terephthalate oven plastic bag (Toppits^®^; Melitta, Minden, Germany). A self-packed glass liner containing 50 mg of the molecular absorbent Tenax TA (GLScience, Eindhoven, Netherlands) was inserted into the upper opening of the bag. Ambient charcoal-filtered air was pushed into the bag through a Teflon tube inserted into a small hole in the bottom at a flow rate of 600 mL min ^-1^ and pulled out over the absorbent at 400 mL min^-1^. Volatiles were collected for 24 h. At least 10 replications were performed per treatment.

After the collection of VOCs, the aboveground dry weight was measured by cutting all plants per pot above the soil, drying them for 72 h at 60°C, and weighing them afterward. Volatile samples were analyzed using gas chromatography/mass spectrometry (GC-MS). The sampling tubes were inserted in an Optic 3 Injector (GLScience, Eindhoven, Netherlands), which was heated from 40°C up to 250°C at 30°C/sec to release the volatiles from the absorbent. Helium was used as a carrier gas (Helium 6.0) with a flow of 1.3 mL min ^1^. The thermal desorbed compounds were separated using an Agilent 7890 N GC system equipped with an HP-1MS capillary column (30 × 0.25 mm inner diameter × 0.25 μm film thickness, 100% dimethylpolysiloxane) and coupled with an Agilent 5975C mass spectrometer (Agilent Technologies, Inc., Santa Clara, CA, USA). The GC temperature program was as follows: Initial oven temperature of 30°C was held for 2 min, increased at a rate of 5°C min^-1^ to 150°C, followed by an increase at a rate of 10°C min^-1^ to the final temperature of 250°C, and then held for 15 min. The temperature of the MS ion source was maintained at 230°C. The quadrupole mass detector was operated in electron impact (EI) mode at 70 eV. The MS gain was set to 10. All data were obtained by collecting the full-scan mass spectra within the range of 40–500 m z^-1^. Authentic standards of volatile compounds for identification were measured under the same GC-MS conditions.

#### 2.3.1 Identification and quantification with AMDIS

Volatile compounds from the chromatograms were identified and quantified using the Automated Mass Spectral Deconvolution and Identification System (AMDIS, V. 2.71; National Institute of Standards and Technology, Boulder, CO, USA). Compound identification was based on the comparison of ion fragmentation patterns and retention indices (RIs) of reference standards. Compounds where no standards were available were annotated as unknowns and their ion fragmentation pattern and RIs were used to ensure the comparison of the same compounds between samples, according to the protocol by Gross et al. (2019). After deconvolution, peak areas were integrated for quantification. Identification and deconvolution criteria were applied as follows: match factor, ≥75%; relative retention index deviation, ≤5% from the reference value; match factor penalties level, very strong; maximum penalty, 20; component width, 12; adjacent peak subtraction, 1; resolution, low; sensitivity, very low; and shape requirements, high. Components with a signal-to-noise ratio of <300 were excluded from the analysis.

#### 2.3.2 Statistical analysis

The overall volatile composition of different wheat monocultures and wheat cultivar mixtures as well as the amounts of individual volatiles were compared using multivariate analysis in R (R Core Team, 2021).

To compare the volatile composition, a Bray–Curtis dissimilarity matrix was calculated using the vegdist function from the ‘vegan’ package (Oksanen et al., 2022). Permutational multivariate analysis of variance (PERMANOVA) of the dissimilarity matrix was calculated using the adonis2 function (N permutations = 10.000). This was followed by pairwise comparisons between wheat monocultures and mixtures using the pairwise.perm.manova function of the ‘RVAideMemoire’ package (Hervé et al., 2022). P-values were adjusted using the Bonferroni method.

The dissimilarities between the VOC profiles of wheat treatments were visualized with a non-metric multidimensional scaling (NMDS) plot generated with the metaMDS function (‘vegan’). We used two dimensions (k = 2) and Wisconsin’s double standardization for scaling.

The amount of single volatiles released by wheat plants was analyzed as the peak area/dry weight (g) of aboveground biomass. Kruskal–Wallis rank sum tests were performed to determine the significant differences in the release of single volatiles between wheat treatments. Pairwise comparisons were performed using Dunn’s test. P-values were adjusted using the Bonferroni method.

## 3 Results

### 3.1 Olfactometer test

**Figure 2:**
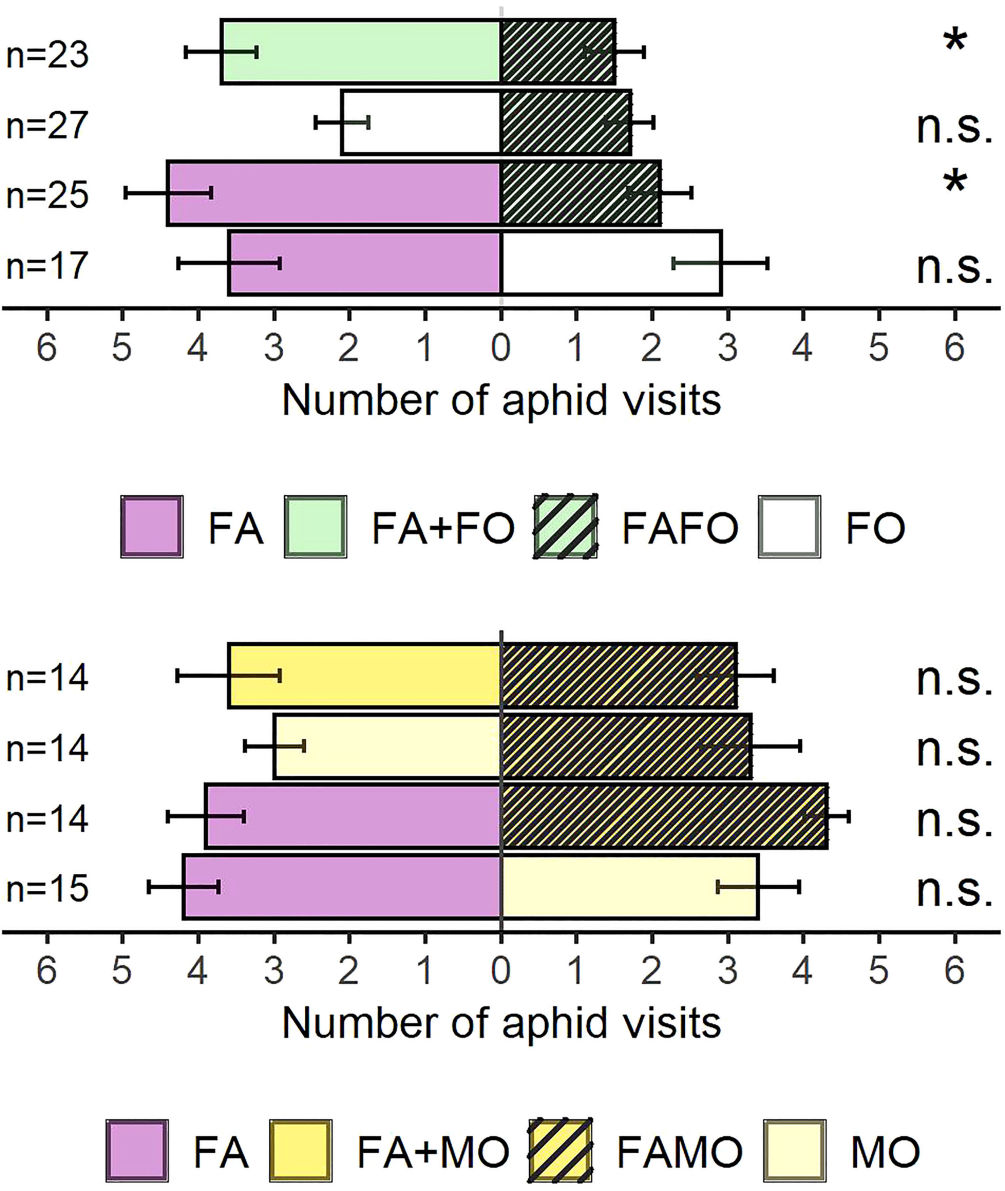
Number of aphid visits to wheat plant odor cues. Comparisons related to FAFO mixture: FA + FO (odor mixture of Florence-Aurora and Forment cultivars when grown in monoculture) vs. FAFO (Florence-Aurora and Forment mixture) (n = 23), FO (Forment monoculture) vs. FAFO (n = 27), FA (Florence-Aurora monoculture) vs. FAFO (n = 25), and FA vs. FO (n = 17). Comparisons related to FAMO mixture: FA + MO (odor mixture of Florence-Aurora and Montcada when grown in monoculture) vs. FAMO (Florence-Aurora and Montcada mixture) (n = 14), MO (Montcada monoculture) vs. FAMO (n = 14), FA vs. FAMO (n = 14), and FA vs. MO (n = 15). Error bars indicate the standard error of the mean. Asterisks indicate significant differences according to the Wilcoxon signed-rank test (P < 0.05).

To identify the effects of the cultivar mixtures on the olfactory response of aphids, olfactometer experiments were conducted with apterous aphids. When the odors of single wheat cultivars grown as monocultures were offered simultaneously, aphids did not show any preference for one of the cultivars (FA vs. FO: Wilcoxon test: Z = 0.67, P = 0.5, n = 17; FA vs. MO: Z = 1.11, P = 0.26, n = 15). Regarding cultivar mixtures, aphids were more attracted to the odor of FA monocultures than to those of FAFO mixtures when offered simultaneously (Z = 2.78, P < 0.01, n = 25) (**Figure 2**). No preference was observed for the FO monoculture odors over those from the FAFO mixture (Z = 0.88, P = 0.38, n = 27) (**Figure 2**).

**Figure 3:**
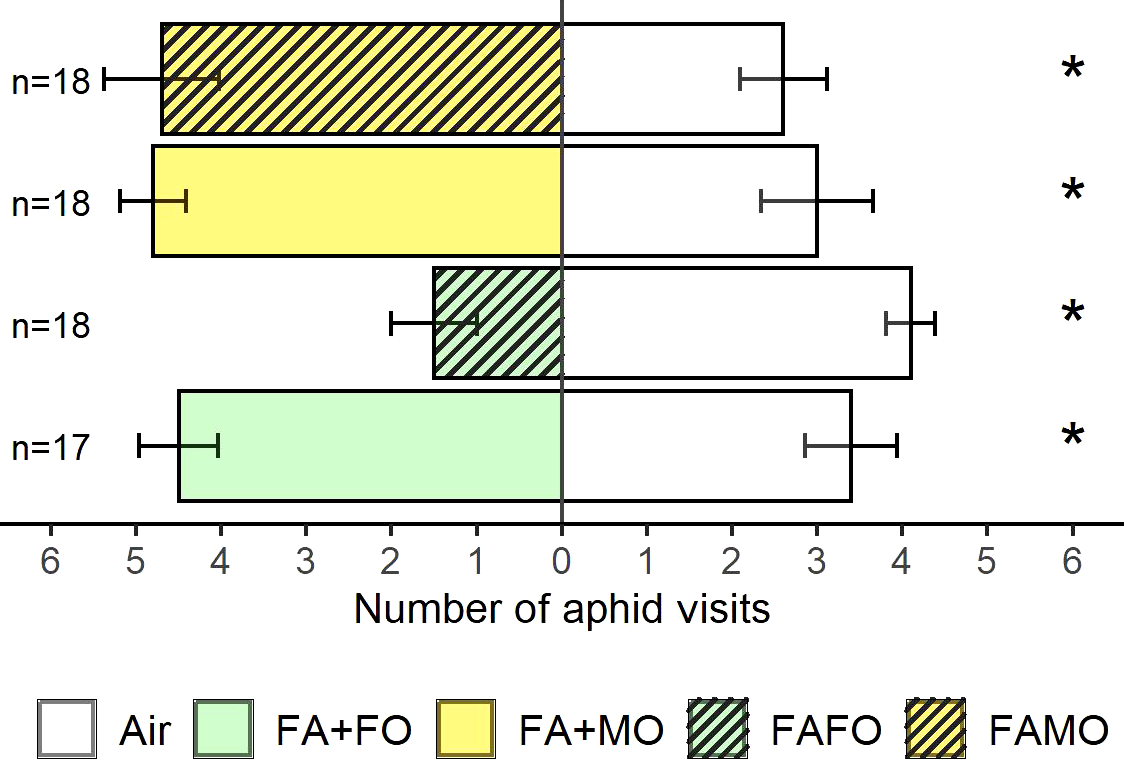
Number of aphid visits to wheat plant odor cues and clean air. Wheat treatments: FA + FO (odor mixture of Florence-Aurora and Forment when grown in monoculture) (n = 17), FA + MO (odor mixture of Florence-Aurora and Montcada when grown in monoculture) (n = 18), FAFO (Florence-Aurora and Forment mixture) (n = 18) and FAMO (Florence-Aurora and Montcada mixture) (n = 18). Error bars indicate the standard error of the mean. Asterisks indicate significant differences according to the Wilcoxon signed-rank test (P < 0.05).

We compared the mixed odors from monocultures (FA + FO and FA + MO) against odors from the cultivar mixture (FAFO and FAMO) to evaluate whether the decrease in attraction for the FAFO mixture odors was due to changes in VOC emission when Florence-Aurora and Forment cultivars grew together or as a mixture of odors from FA and FO. Aphids preferred the odor cues from FA + FO over those from FAFO (Z = 2.72, P < 0.01, n = 23) (**Figure 2**). In contrast, the FAMO mixture did not affect aphid behavioral responses (**Figure 2**). In the avoidance test, FAFO was the only treatment that elicited aphid avoidance (Z = 2.63, P < 0.01, n = 20). As expected, in the remaining treatments, aphids were significantly more attracted to plant odor cues than to clean air: FA + FO (FA + FO vs. air: Z = 2.43, P = 0.05, n = 19), FA + MO (FA + MO vs. air: Z = 2.09, P = 0.05, n = 20), and FAMO (FAMO vs. air: Z = 2.31, P = 0.05, n = 20) (**Figure 3**).

### 3.2 VOCs profile

To investigate the impact of intraspecific interactions between wheat cultivars on the volatile emission, the headspace of wheat plants of three cultivars grown as monocultures or in a two-way mixture was sampled and analyzed via TD-GC-MS.

**Figure 4:**
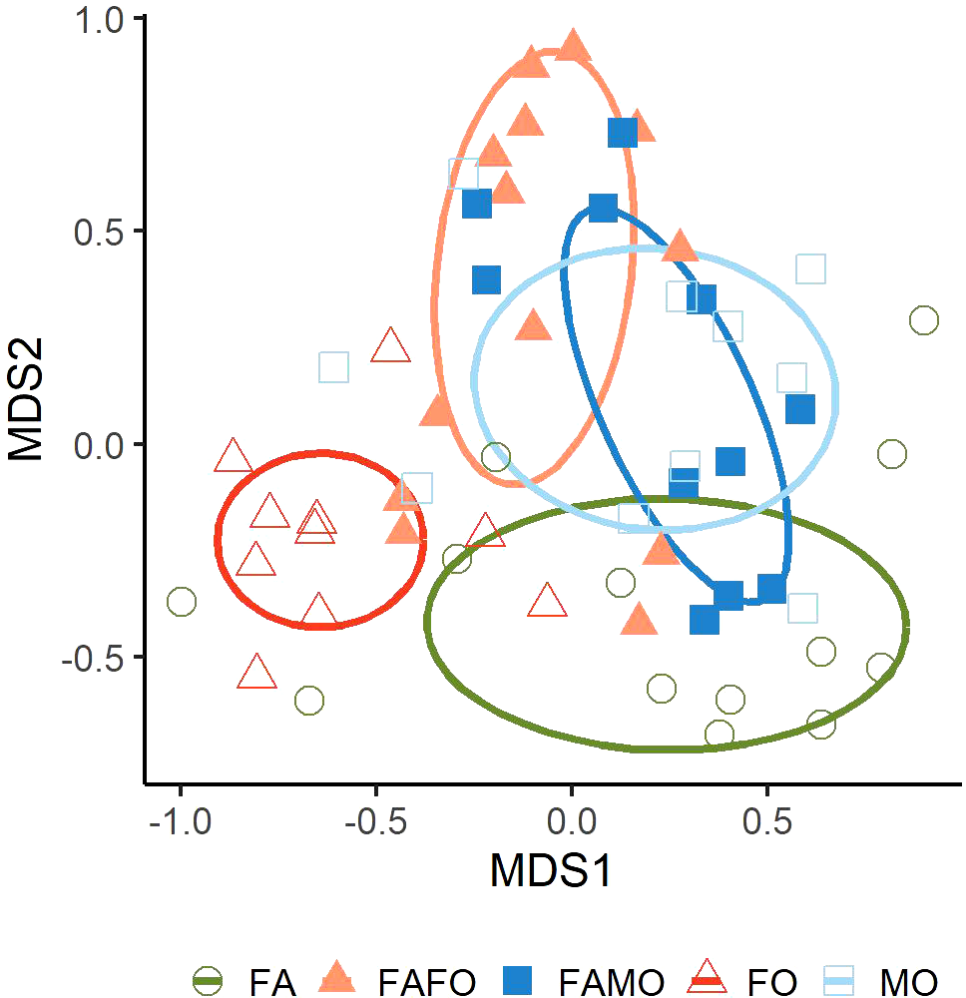
Non-metric multidimensional scaling (NMDS) plot illustrating the Bray-Curtis dissimilarities of the proportional volatiles compositions of wheat cultivars Florence-Aurora (FA) (n = 13), Forment (FO) (n = 10) and Montcada (MO) (n = 10) grown in monocultures and in mixtures: Florence-Aurora with Forment (FAFO) (n = 13) and Florence-Aurora with Montcada (FAMO) (n = 11).

We analyzed the composition of 88 detected compounds emitted by wheat plants. The volatile compositions differed significantly between cultivars in monocultures and mixtures (PERMANOVA, df = 4, R^2^ = 76.13, N = 10.000, P < 0.001). Pairwise comparisons showed significant differences in odor profiles of Florence-Aurora and Forment in monoculture (P < 0.01) as well as compared to the FAFO mixture (**Figure 4**). The odor compositions of the FAMO mixture were not distinguishable from the odor profiles of FA and MO monocultures (**Figure 4**).

**Figure 5:**
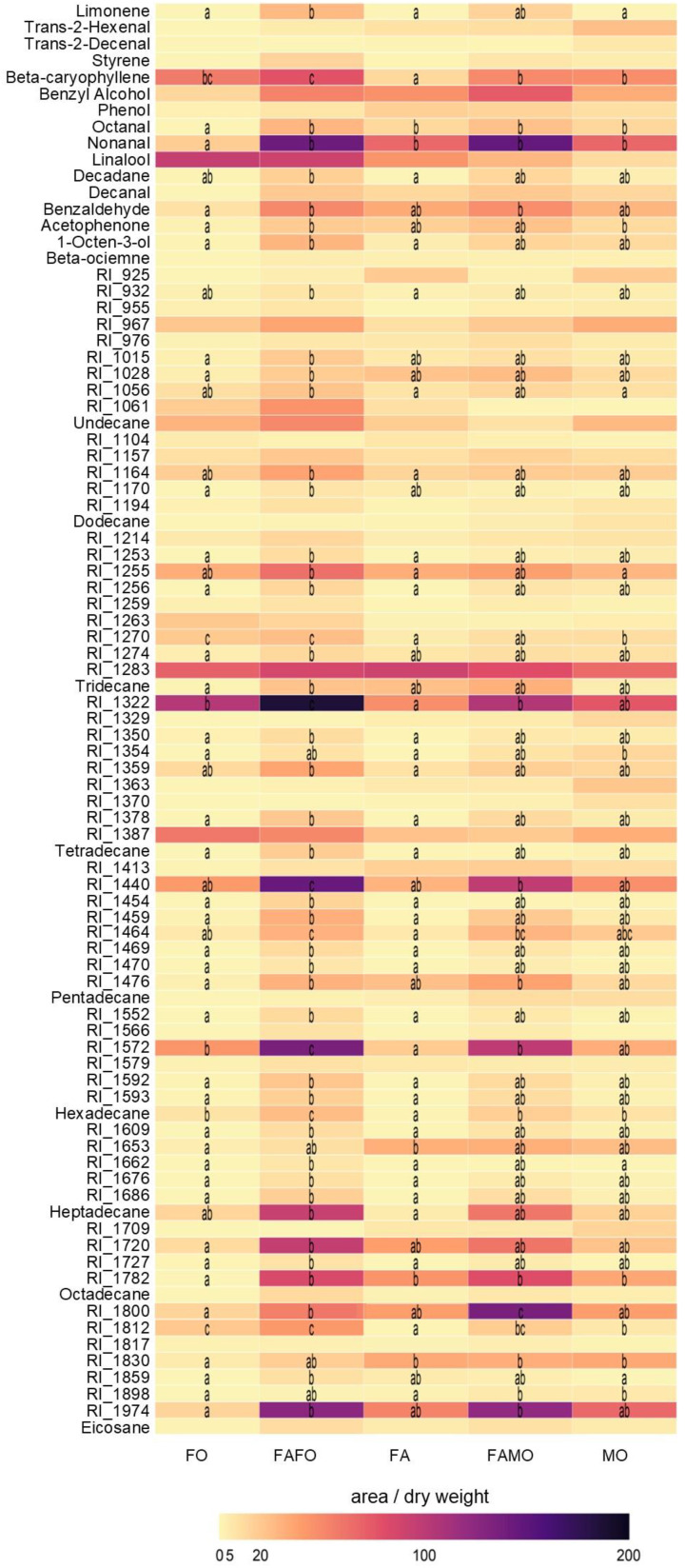
Composition of the volatile chemicals obtained by GC/SM of wheat treatments: Florence-Aurora (FA) (n = 13), Forment (FO) (n = 10), Montcada (MO) (n = 10), Florence-Aurora and Forment mixture (FAFO) (n = 13), and Florence-Aurora and Montcada mixture (FAMO) (n = 11). Dark blue indicates a high relative abundance of a respective volatile compound, and light yellow a low abundance. Numbers are mean values of compound abundance (compound peak area/dry weight (g)). Letters indicate significant differences according to Dunn´s pairwise test (P < 0.05).

Overall, cultivar mixtures released higher amounts of VOCs than the three monocultures (**Figure 5**). Particularly, FAFO emitted significantly higher amounts of 33 specific compounds when compared to FA (37.5%) and of 40 compounds when compared to FO (45.4%), including β-caryophyllene, β-ocimene, limonene, 1-octen-3-ol, nonanal, octanal, benzaldehyde, acetophenone among other unknown compounds (**Figure 5**). VOC emission from the FAMO mixture was similar to that from the FA and MO monocultures, only releasing a significantly higher amount of eight (9.09%) and two (2.27%) compounds, respectively.

## 4 Discussion

### 4.1 Aphid olfactory response to cultivar mixtures

The purpose of this study was to assess whether combining wheat cultivars can modify the volatile mixture profile and, consequently, the aphid host-location response. Aphids are extremely sensitive to slight changes in their hosts’ odor cues, which they utilize as host-finding signals (Webster, 2012). Therefore, the alterations in mixture odor cues caused by plant-plant interactions can influence the aphid host-locating response and its attractiveness to particular wheat combinations, as demonstrated by our mixture of Florence-Aurora and Forment in olfactometer experiments with *S. avenae*. Moreover, the volatiles emitted by Florence-Aurora with the formation mixture led to avoidance by *S. avenae*. Our results indicated that the Florence-Aurora and Forment mixture prevented aphid host localization by releasing non-attractive olfactory signals, thereby reducing aphid acceptance and further aphid infestation. Studies have demonstrated the importance of host-specific volatile compounds and their ratios in the overall composition of aphid host-location behavior (Webster, 2012). For instance, regarding the volatile ratio in olfactometer tests reproducing hop (*Humulus lupulus*) leaf volatile cues, Campbell et al. (1993) found that aphids responded positively to the odor of (E)-2-hexenal and β-caryophyllene in an approximate ratio of 39:1 by weight. However, when the ratio was adjusted to 1:1, no response was observed. Therefore, a shift in the odor cues of Florence-Aurora and Forment cultivars when grown together may influence plant-pest interactions by decreasing crop odor attractiveness to aphids. In contrast, mixing Florence-Aurora and Montcada did not affect the aphids’ host-locating behavior compared to the monocultures or the odor mixture of the two cultivar monocultures, which is consistent with the absence of volatile profile alterations shown in the mixture.

### 4.2 Volatile emission of cultivar mixture

In this study, we assessed the interactions of undamaged wheat cultivars Florence-Aurora with Forment and Florence-Aurora with Montcada when grown together. The results showed that only certain cultivar combinations induced physiological responses to the volatiles emitted when grown together, suggesting the specificity of the effect of genotypic diversity on aphid control. Corroborating with previous experiments, headspace analysis revealed a higher amount of single volatile compounds released from the wheat cultivar mixture than from the monocultures (Shoffner and Tooker, 2013).

Airborne volatiles are crucial signals for inter- and intraspecific plant-plant interactions. Plants constantly emit VOCs and, in return, are constantly exposed to VOCs from damaged and undamaged neighboring plants (Callaway, 2002). These odor cues from emitter plants can affect complex biochemical pathways in the receiver plants (Midzi et al., 2022). Although most studies have focused on the induced defense response in plants receiving VOCs from herbivore-attacked plants (Hu et al., 2019; Midzi et al., 2022), previous studies have demonstrated that VOCs from undamaged plants also trigger morphological and physiological responses in receiver plants (Ninkovic et al., 2016; Kheam et al., 2023). For example, plants of the barley cultivar Kara (*Hordeum vulgare*) allocate more biomass to their roots after exposure to VOCs from cv. Alva compared to the unexposed plants or cv. Kara plants previously exposed to VOCs of another Kara plant (Ninkovic et al., 2003).

We observed physiological responses in the form of altered release of VOCs by the Florence-Aurora and Forment cultivars when mixed. Furthermore, the analysis of specific compound amounts demonstrated that Florence-Aurora and Forment interactions, when grown together, altered the emitted amount of certain compounds, shifting the volatile ratio, which plays an important role in aphid host location (Webster, 2012). Regarding specific volatile chemicals, Visser and Fu-shun (1995) demonstrated that *S. avenae* was attracted to 2-hexanal, benzaldehyde, and linalool, but vaguely responded to 1-octen-3-ol, β-caryophyllene, and limonene odor signals. Moreover, 2-hexanal, linalool, octanal, nonanal, and caryophyllene are assumed to be strong cereal aphid attractants *S. avenae* and *Rhopalosiphum padi* (Linnaeus) (Pickett et al., 1997; Quiroz and Niemeyer, 1998; Birkett et al., 2010). In our study, the abundance of these volatile chemicals was greatest in the Florence-Aurora and Forment mixture, whose odor cues were surprisingly less attractive than those of the monocultures. This suggests that the plant volatile ratio may play a more important role in modifying aphid behavior than the abundance of specific volatiles (Bruce and Pickett, 2011).

Further, the analysis of the odor profile of single cultivars confirmed that the Florence-Aurora cultivar had a volatile profile similar to that of the Montcada cultivar but significantly different from the Forment profile. The interaction between the Florence-Aurora and Montcada cultivars did not affect the mixed odor profile. In line with previous volatile barley experiments, our findings support the hypothesis that greater differences between cultivars’ odor profiles might induce greater physiological responses (Dahlin et al., 2018).

In conclusion, our study supports the “right neighbor” hypothesis by demonstrating an intraspecific interaction effect on the odor profile of mixtures, exclusive to certain cultivar mixtures, and might explain the dependence of genotypic diversity on aphid control recorded in numerous field studies (Ninkovic et al., 2002; Dahlin et al., 2018). Our results suggest that the similarity/dissimilarity of VOC emissions is important for plant-plant interactions between the combined cultivars. In the present study, wheat cultivars with distinct profiles affected each other, resulting in an odor that was less attractive to *S. avenae*. Future studies should address whether plants with generally dissimilar odor profiles are more likely to have an impact on each other, and thereby possibly promote associational resistance and enhance aphid control.

## Data availability statement

The raw data supporting the conclusions of this article will be made available by the authors, without undue reservation.

## Author contributions

VN, JG, FS, LC-L, and AT-F conceived and designed the research. JG, AT-F, and AE conducted the experiments. JG and AT-F statistically analyzed the data. AT-F wrote the first draft of the manuscript. VN and JG reviewed, commented, and polished the manuscript. All authors contributed to the article and approved the submitted version.
